# Parents' Knowledge and Perception Toward Short Stature in Saudi Arabia

**DOI:** 10.7759/cureus.51163

**Published:** 2023-12-27

**Authors:** Kadi A Alhumaidi, Eman A Alotaibi, Salman Almansour, Aeshah Alharbi, Norah H Alharbi, Shahad M AlJameli, Ghadah A Aljateli, Njood M Alobaid, Reema A Almasoud

**Affiliations:** 1 Medicine, Unaizah College of Medicine and Medical Sciences, Qassim University, Unaizah, SAU; 2 Family and Community Medicine, Unaizah College of Medicine and Medical Sciences, Qassim University, Unaizah, SAU; 3 Pediatric Endocrinology, Qassim University, Qassim, SAU; 4 Medicine, Unaizah College of Medicine and Medical Sciences, Qassim University, Qassim, SAU; 5 College of Medicine, King Saud University, Riyadh, SAU

**Keywords:** parents' knowledge, saudi arabia, family medcine, pediatric endocrine, short stature

## Abstract

Introduction

Short stature is a common reason for referral to pediatric endocrinologists. A Saudi study highlights significant short stature prevalence, with parents exhibiting varied knowledge levels. Common normal variants of short stature are familial short stature, constitutional, and idiopathic short stature. Pathologic causes of short stature include growth hormone deficiency, genetic disorders, and chronic diseases. Parents' knowledge plays an important role in the diagnosis and early intervention of this condition. Insufficient studies prompt the authors to conduct a novel survey assessing Saudi parents' knowledge and perceptions of short stature, filling a research gap.

Methodology

This is a cross-sectional study conducted among Saudi Parents in five different regions of Saudi Arabia. A self-administered questionnaire was distributed among parents via an online survey. The questionnaire includes sociodemographic characteristics and questions to assess the knowledge and perception regarding short stature. Non-probability sampling targets parents living in Saudi Arabia. Data is analyzed by SPSS version 29 (IBM Inc., Armonk, New York).

Results

Our study on Saudi parents' knowledge of short stature reveals diverse awareness levels. While genetic causes are widely recognized in (71.6%; N=245) of parents (N=352), awareness drops for factors like low birth weight (23.9%; N=82) total of (N=352). Parents show uncertainty in recognizing short stature (51.4%; N=352) and varied beliefs on growth cessation. A majority (65.6%; N=231) of parents (N=352) prefer early intervention, with 41.5% (N=146) of parents (N=352) recognizing growth hormone therapy. Sociodemographic factors influence knowledge scores, with higher scores in males (21.03) and Central region residents (22.03; p<0.001). Notably, 83.4% (N=248) of parents (N=352) acknowledge psychological complications.

Conclusion

Our study highlights varied awareness among parents regarding short stature, emphasizing genetic causes but demonstrating gaps in recognizing certain factors. Sociodemographic factors significantly influence knowledge scores. Psychological complications are widely acknowledged.

## Introduction

Short stature is characterized by a child's height being within or below the third percentile of the mean height for the age, sex, and population group. It can be measured using a variety of tools, including measuring tapes, stadiometers, anthropometric rods, infantometers, etc. Depending on the cause, short stature has been referred to by a variety of names [1].

Short stature is a hereditary trait and is controlled by both genetic as well as environmental factors [2]. It has four main causes: genetic, constitutional growth delay, early puberty, and medical reasons such as endocrine, bone, genetic, and chronic disorders [3-6].

There is a greater chance that the children will be short-statured if their parents are short or if it runs in their family. But, hereditary short stature is only relevant in cases when there is no underlying medical condition. This is also known as inherited short stature. [3].Those with low stature due to their genetic makeup will grow to a height within the desired height range. They grow normally and do not experience a delay in the onset of bone aging [4] despite the fact that familial (genetic) short stature, which accounts for most of the cases, is a normal non-pathologic variant of growth. Individuals with familial short stature experience various forms of physical and psychological stress in modern society. Children are frequently referred to endocrinology clinics for treatment because of their short stature [7].

Moreover, constitutional growth delay deals with the velocity of growth. The growth process of these individuals may be slow or normal. But they typically catch up as adults, having had short stature as children but rather normal heights as adults. Malnutrition during pregnancy and infancy, as well as genetic factors, could be the reason behind having short stature. One factor affecting the rate of growth and bone development is malnutrition, which can exacerbate short stature in a person who is genetically susceptible to have short stature [5].

It's important to diagnose and treat children who suffer from short stature. Preventable illnesses can considerably improve, and their growth can be accelerated to catch up with their peers with early diagnosis and treatment [8]. The main goal of treating short stature should be to address the underlying cause. Hormonal therapy should be utilized to address short stature brought on by an underlying hormone deficit. Moreover, it is important to address the underlying bone disease that causes short stature. The hormonal disorder that causes short stature can be treated today with a variety of hormonal medications, which should be provided as soon as feasible. This will address the underlying issue, stop short stature from developing, and stop its psychosocial repercussions. They include using gonadotropin-releasing hormone analogs (GnRHa), aromatase inhibitors, recombinant human insulin-like growth factor-1 (RhIGF-1), low-dose androgen therapy, and recombinant human growth hormone to treat growth hormonal deficits and congenital growth delays (rhGH)[6]. 

A 2004-2005 study in Saudi Arabia that included 19,372 healthy children and adolescents aged 5 to 17 years found that a significant portion of the population had short stature. For example, the researchers reported that the prevalence of short stature in boys (N=19,372) was 1.8% in adolescents and 11.3% in children, whereas they discovered that it was 1.2% in girls and 10.5% in children [9]. A study was conducted in the Qassim region to see how Saudi parents felt about short stature and its effects. They discovered that 63.5% of respondents (N=384) knew that a child's short stature was determined by careful inspections and calculations made by an attending physician. Parents were also aware that seeking medical advice is required when short stature is noted. Overall, the results showed that 68.4% of the population (N=384) investigated using random samples had poor knowledge levels, while 31.6% had good knowledge levels, with a total knowledge score (mean (SD)) of 2.04 (0.92). The following contributed to high knowledge levels: having children, being married, and having a history of short stature in the family. Just 26% of respondents (N=384) thought drug therapy was the best intervention for short-statured children, whereas the majority (68%) of total parents chose both therapeutic and non-therapeutic measures, demonstrating the Saudi parents' knowledge of intervention and treatment alternatives. We think that the well-known physiotherapeutic techniques and exercises for people with short stature are what led to this outcome [7].

While we were researching this topic, we noticed insufficient studies assessing the parents' knowledge and perception toward short stature in Saudi Arabia. In our study, we aim to assess it by developing a survey that targets Saudi parents.

## Materials and methods

The study was conducted as an observational, cross-sectional study from 6 February 2023 to 6 December 2023, targeting the parents through an online survey that was distributed through four regions of Saudi Arabia (Central, Northern, Western, and Southern). The sampling technique was non-probability sampling, with inclusion criteria being parents who are living in Saudi Arabia while exclusion criteria were parents who are living outside Saudi Arabia.

The data were collected using a structured questionnaire consisting of five sections. The first section included sociodemographic factors. The second section included questions to assess the knowledge about the diagnosis of short stature. The third section included questions to assess knowledge about the causes of short stature. The fourth section included questions to assess knowledge about the treatment options for short stature. The final section included questions to assess the knowledge about the complications of short stature.

A pilot or preliminary study on 15% of the participants was done as a pre-test for our research instrument to ascertain any issues and barriers in recruiting participants. The data from the pilot study were not included in the main study.

Data were analyzed using SPSS version 29 (IBM Inc., Armonk, New York), with qualitative data expressed as numbers and percentages and quantitative data expressed as mean and standard deviation. The chi-square (x2) test was used to assess the relationship between two or more qualitative variables. The Student t-test was used for comparing two quantitative normally distributed variables, and the ANOVA test was used for comparing more than two quantitative normally distributed variables with the significant level set at p-value <0.05. 

Ethical approval (23-52-01) was received from the Regional Ethical Committee of Qassim Region, the Kingdom of Saudi Arabia, and participants were ensured confidentiality and the freedom to withdraw from the study at any time.

## Results

Our study included 352 parents assessed for knowledge about short stature. Table 1 shows that the majority were females (53.7%; N=189), with males comprising 46.3% (N=163) of parents. Age distribution showed prevalence in the 30-39 years group (28.7%; N=101). Education levels varied, with 52.8% (N=186) holding a bachelor's degree. Most of the participants were Saudi (98.3%; N=346), and regionally, the Central region had the highest representation (31.5%; N=111).

**Table 1:** Sociodemographic and other parameters of parents

Variable		Frequency (n=352)	Percent
Gender	Females	189	53.7
	Males	163	46.3
Age	<20 Years	17	4.8
	20-29 Years	66	18.8
	30-39 Years	101	28.7
	40-49 Years	92	26.1
	50-59 Years	55	15.6
	>60 Years	21	6.0
Education	Primary	30	8.5
	Secondary	99	28.1
	Bachelor's	186	52.8
	Higher education	37	10.5
Nationality	Non-Saudi	6	1.7
	Saudi	346	98.3
Region	South	95	27.0
	North	75	21.3
	Western	71	20.2
	Central	111	31.5

**Figure 1:**
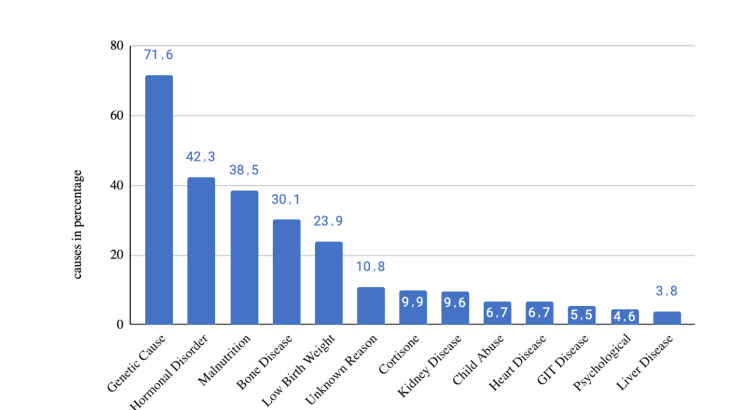
Parents' knowledge about the causes of short stature GIT - gastrointesntinal

Figure 1 shows parents' knowledge regarding the causes of short stature. Genetic causes are widely recognized, with 71.6% (N=245) of participants acknowledging this factor. Hormonal disorders (42.3%; N=145), malnutrition (38.5%; N=131), and bone diseases (30.1%; N=103) are also identified contributors. However, awareness drops for factors like low birth weight (23.9%; N=82), unknown reasons (10.8%; N=37), and specific medications like cortisone (9.9%; N=34). Less commonly recognized factors include kidney disease, child abuse, heart disease, gastrointestinal disease, psychological disease, and liver disease, emphasizing varied awareness levels among parents regarding short stature causes.

Table 2 shows a comprehensive overview of parents' knowledge and perceptions regarding the diagnosis, treatment, and complications of short stature among children. A significant portion of parents (51.4%; N=181) of is unsure about recognizing short stature when a child is 3 to 5 percent shorter than peers. Furthermore, a substantial number (67.9%; N=239) is unfamiliar with the Saudi growth chart. Regarding the criteria for identifying short stature, responses vary, with a notable percentage (33.2%) considering a child to have a short stature if they are shorter than their class colleagues. Regarding the ideal timing for medical intervention, a significant majority (65.6%; N=231) believe it's best to consult a doctor as soon as they notice short stature or before puberty. In terms of intervention nature, growth hormone therapy is well-recognized by 41.5%(N=146), with an additional 46.0%(N=162) acknowledging the importance of treating the underlying cause. The route of growth hormone administration varies, with 32.4%(N=114) opting for intramuscular injection. In terms of medical expertise, 40.9% (N=144) recognize pediatric endocrinology as the most qualified field for consultation on children's short stature. Lastly, awareness of complications associated with short stature is notable, with 83.4% (N=248) acknowledging psychological complications in adolescence, indicating a comprehensive understanding beyond physical aspects.

**Table 2:** Knowledge and perception of parents about diagnosis, treatment, and complications of short stature

Knowledge about diagnosis	Answers	Frequency (n=352)	Percent
Child considered to have a short stature if the height of a child is 3 to 5 percent shorter than that of children of the same age and sex?	Don't Know	181	51.4
	No	52	14.8
	Yes	119	33.8
Child considered to have a short stature if the child is shorter than his/her class colleagues?	Don't Know	111	31.5
	No	124	35.2
	Yes	117	33.2
Child considered to have a short stature only when the doctor examines the child and proves the short stature by calculations?	Don't Know	118	33.5
	No	64	18.2
	Yes	170	48.3
Are you familiar with the Saudi growth chart (picture)?	No	239	67.9
	Yes	113	32.1
What is a normal growth rate for a children aged four years till puberty per year?	1-3 cm	48	13.6
	3-6 cm	112	31.8
	6-8 cm	120	34.1
	>8 cm	72	20.5
At what age does the normal growth of children stop both in boys and girls?	Not fixed	73	20.7
	12 years	31	8.8
	12-15 years	18	5.1
	12-18 years	95	27
	Up to 20 years	135	38.4
Knowledge about treatment			
When is the ideal time to see a doctor about short stature?	When child complains	55	15.6
	After puberty	66	18.8
	As soon as notices/before puberty	231	65.6
What is the nature of intervention for short-statured children?	Surgical treatment	44	12.5
	Growth hormone therapy	146	41.5
	Treat underlying cause	162	46
What is the route used to administer the growth hormone?	Mouth	109	31
	IV injection	87	24.7
	IM injection	114	32.4
	Subcutaneous	42	11.9
For a consultation on children's short stature, what medical field is the most qualified?	Internal medicine	67	19
	General pediatrics	136	38.6
	Pediatric endocrinology	144	40.9
	Other	5	1.4
Knowledge about complications			
Complications of short stature	Delayed sexual development	130	38.1
	Difficult childbirth for females in the future	140	40.9
	Psychological complications in adolescence	284	83.4

**Figure 2:**
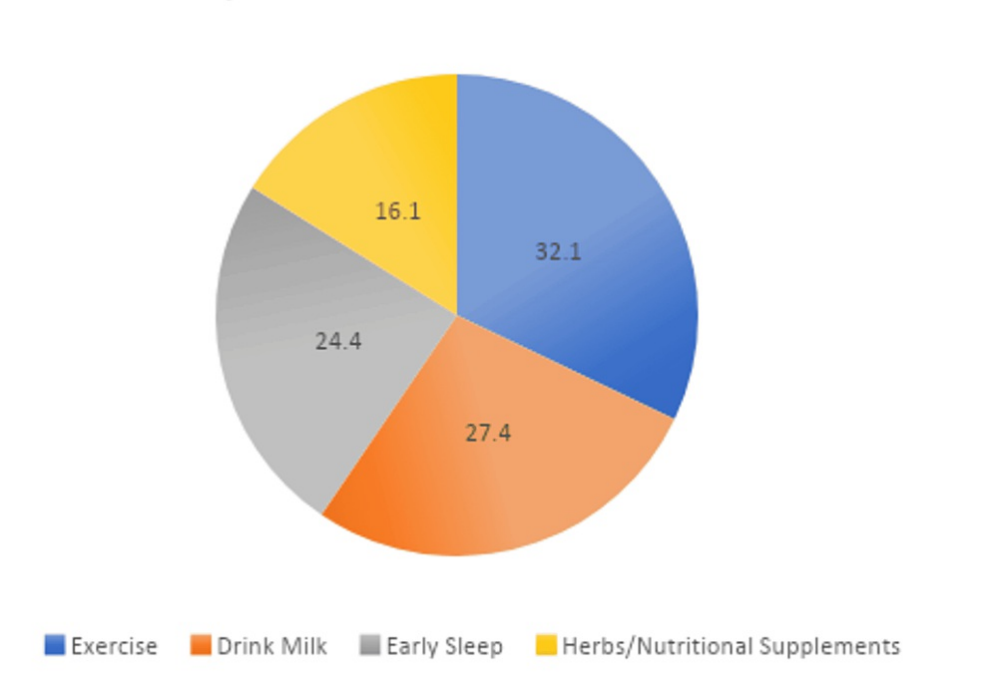
Non-therapeutic interventions of short stature according to parent's perception

Figure 2 shows parents' perceptions of non-therapeutic interventions for short stature. Notably, 32.1% of parents believe that exercise is a non-therapeutic measure to address short stature. Drinking milk is perceived as such by 27.4% (N=175) of parents, while 24.4% consider early sleep as a non-therapeutic intervention. Additionally, a portion of parents (16.1%) believe in the effectiveness of herbs and nutritional supplements as non-therapeutic measures for addressing short stature.

**Figure 3:**
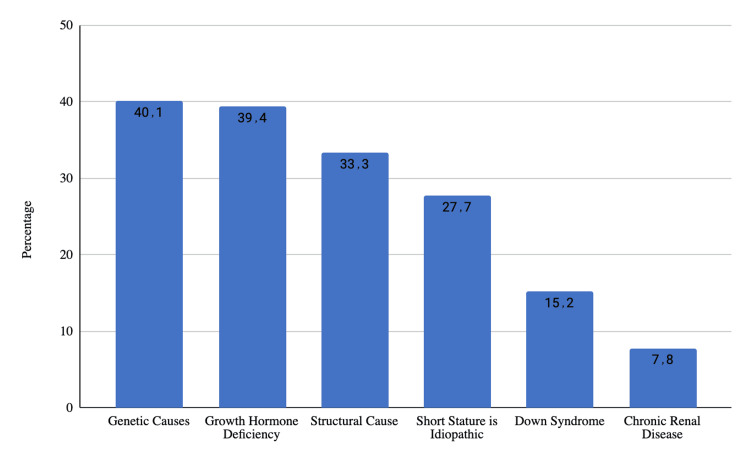
Parents' knowledge about the candidates of growth hormone therapy for short stature

Figure 3 shows parents' knowledge about the candidates for growth hormone therapy for short stature. The majority (40.1%) of parents recognize genetic causes as candidates for growth hormone therapy. Close behind, 39.4% acknowledge growth hormone deficiency as a relevant factor. Structural causes are identified by 33.3% of parents, while 27.7% understand that short stature can be idiopathic. Specific conditions like Down syndrome (15.2%) and chronic renal disease (7.8%) are also perceived as candidates for growth hormone therapy, though to a lesser extent.

Table 3 shows the association between sociodemographic features and Saudi parents' knowledge scores about short stature. Notable trends include higher knowledge scores in males (21.03) compared to females (20.52), which is statistically significant (p<0.001). There is a non-significant difference across age groups, with parents aged 40-49 years exhibiting the highest mean score (21.76). Education levels do not significantly impact scores. While non-Saudi parents show a higher mean score (23.00), the difference is not statistically significant. Region-wise, parents in the Central region have the highest mean knowledge score (22.03), while those in the South score the lowest (19.03), which is statistically significant (p<0.001).

**Table 3:** Association of different sociodemographic features with the high knowledge score of parents about short stature

Variable		Saudi parents' knowledge score about short stature			
		N	Mean	Std. deviation	Sig. value
Gender	Females	17	20.52	2.91	<0.001
	Males	66	21.03	3.01	
Age	<20 Years	101	20.77	3.19	0.222
	20-29 Years	92	20.89	3.31	
	30-39 Years	55	19.72	3.16	
	40-49 Years	21	21.76	3.25	
	50-59 Years	189	21.69	2.79	
	>60 Years	163	19.62	3.28	
Education	Primary	30	20.00	2.86	0.595
	Secondary	99	20.59	3.13	
	Bachelor's	186	20.86	3.28	
	Higher education	37	21.05	3.19	
Nationality	Non-Saudi	6	23.00	2.75	0.065
	Saudi	346	20.69	3.19	
Regions	South	95	19.03	3.06	<0.001
	North	75	19.88	3.13	
	Western	71	21.88	2.89	
	Central	111	22.03	2.66	

## Discussion

Short stature, measured within the third percentile, results from genetic, environmental, and medical factors. Ali et al. and Barstow et al. defined short stature (as height >2 standard deviations (SD) below the mean for age) and growth failure (as a subnormal height velocity that leads to a decline in growth percentiles, usually height velocity >1.5 SD below the mean for age) are common pediatric concerns [10,11]. Genetic causes, including familial short stature, lead to physical and psychological stress. Constitutional growth delay may cause slow growth, while malnutrition and hormonal deficits are significant medical factors. Treatment aims to address underlying causes by utilizing hormonal therapies. Similarly, Rani et al. show the primary management of short stature aims at alleviating the underlying cause [12]. A Saudi study highlighted a significant prevalence of short stature in children. However, a survey revealed insufficient studies on Saudi parents' knowledge. Our study intends to fill this gap, assessing parental perception in a region lacking prior research. Our study findings shed light on crucial aspects such as awareness of causes, diagnostic criteria, treatment options, and sociodemographic influences on parental knowledge scores.

Our results highlight a prevalent awareness of genetic causes (71.6%; N=245) among parents, indicating a foundational understanding of the hereditary factors contributing to short stature. This aligns with existing literature that emphasizes the significance of genetic factors in determining height. Jee et al. show that short stature is a common and heterogeneous condition that is often genetic in etiology [13]. Moreover, the SHOX gene, first described in 1997 by Rao et al., has been implicated as an important cause of short stature [14,15]. However, a notable gap exists in recognizing other contributing factors, such as hormonal disorders, malnutrition, and bone diseases, where awareness varies. Common endocrine disorders, described by Rabbani et al., leading to short stature include hypothyroidism, Cushing's syndrome, and growth hormone deficiency [16]. The lower recognition of specific factors like low birth weight and cortisone medication suggests a need for targeted education to enhance parental understanding of diverse causative elements.

Moreover, there are diverse responses regarding diagnostic criteria for short stature revealing a lack of consensus among parents. A substantial portion (51.4%) is uncertain about recognizing short stature based on a three to five percent difference in height compared to peers. Similarly, an article from Johns Hopkins University confirms that short stature typically means that a person's height is below that of the shortest three to five percent of children of the same age and sex [17]. Additionally, a majority (67.9%) are unfamiliar with the Saudi growth chart, an essential tool for assessing a child's growth trajectory. In contrast, Nahedh et al. show that in Saudi Arabia, 35.8% of mothers were aware of the growth chart [18]. Varied perceptions of when normal growth stops (38.4% believing it continues up to 20 years) further highlight the need for standardized educational interventions to align parental understanding with medical guidelines.

Parents exhibit significant awareness of the importance of early medical intervention for short stature, with 65.6% advocating consultation as soon as they notice the condition or before puberty. This aligns with medical recommendations emphasizing early detection for effective intervention. Moreover, Kaplan et al. show that early detection of abnormal growth in childhood allows for timely intervention that may prevent excessive short stature in adulthood [19]. Notably, growth hormone therapy is recognized by 41.5% of parents, indicating a reasonable level of awareness. However, there is a substantial percentage (46.0%) acknowledging the importance of treating the underlying cause, emphasizing a comprehensive understanding of treatment modalities beyond hormone therapy.

There is variation in routes for growth hormone administration, with 32.4% of parents answering intramuscular injection as the correct route to administer the growth hormone, suggesting diverse perspectives on treatment methods. Additionally, recognizing pediatric endocrinology as the most qualified medical field for consultation (40.9%) is encouraging, as it indicates an understanding of the specialized care needed to address short stature.

Parents' perceptions of non-therapeutic interventions reveal interesting insights. Exercise is commonly perceived as a non-therapeutic measure by 32.1% of parents, indicating a belief in the potential role of lifestyle factors in managing short stature. Similarly, Sun et al. show that regular and moderate stretching exercises combined with lysine-inositol vitamin B12 can effectively promote height growth of children with idiopathic short stature (ISS), which is clinically safe [20]. Drinking milk (27.4%) and early sleep (24.4%) are also recognized as non-therapeutic measures, reflecting a holistic approach to child health. The acknowledgment of these non-medical interventions suggests a multifaceted understanding among parents, encompassing both medical and lifestyle factors.

Parents' recognition of genetic causes (40.1%) and growth hormone deficiency (39.4%) as candidates for growth hormone therapy aligns with medical literature. LaFranchi et al. stated that the accepted indication for human growth hormone (hGH) treatment is congenital classic human growth hormone (hGH) deficiency [21]. However, the awareness of structural causes (33.3%) and the understanding that short stature can be idiopathic (27.7%) indicates a nuanced awareness of the complexity of factors influencing eligibility for growth hormone therapy. Specific conditions like chronic renal disease are also recognized, albeit to a lesser extent.

There is an association between sociodemographic features and parents' knowledge scores, which provides valuable insights. While gender and region significantly influence knowledge scores, other factors such as age, education, and nationality show non-significant associations. The higher knowledge scores in males suggest potential gender-based differences in information access or health-seeking behavior. Region-wise variations highlight the influence of local factors on awareness levels, emphasizing the need for targeted educational initiatives tailored to regional contexts. Hashem et al. show that participants living in the northern and eastern areas were more likely to be knowledgeable about short stature compared to others (p≤0.01) [22].

There are several limitations, which include relying on self-reported data and the online survey may introduce response bias. The cross-sectional design limits establishing causation. Longitudinal studies could explore the dynamic nature of parental knowledge. In-depth qualitative research may uncover nuanced perceptions. Understanding parental knowledge aids in tailored interventions. Addressing gaps can enhance awareness, contributing to better health outcomes for children with short stature.

## Conclusions

Our study provides a comprehensive overview of parents' knowledge and perception of short stature in Saudi Arabia. The findings underscore the need for targeted educational interventions to enhance awareness of specific causes, diagnostic criteria, and treatment options. Understanding sociodemographic influences on knowledge scores is crucial for tailoring educational initiatives to address regional and gender-based variations. Improving parental awareness is not only essential for early intervention but also contributes to fostering a holistic understanding of child health beyond medical interventions.
